# Supplementary material for: Alterations in the inflammatory markers of the Tumor Necrosis Factor system in overweight and obese children and adolescents
Source: PLoS One. 2025 May 13;20(5):e0319832. doi: 10.1371/journal.pone.0319832 (PMC12074418; doi:10.1371/journal.pone.0319832)
Supplement: Attachment — Submitted filename: Response to reviewers.docx [file pone.0319832.s003.docx]

Response to reviewers:

Manuscript title: Alterations in the inflammatory markers of the Tumor Necrosis Factor system in overweight and obese children and adolescents.

Larger comments:

Major comments:
1. Under materials and methods, lines 120-139 are a bit confusing with the grammar. Follow the heading and revise accordingly the entire section. I must the criteria of participant selection is confusing and given slim chances that it can replicated:

Response: In materials and methods, grammar was revised and the article was sent to Wiley Editing Services, as you can see in the certificate that we attached in the supplementary materials. The changes made are marked in the revised text.

Lines 129-139 were written as follows:

From February 2013 to July 2014, 172 children (5 to 9 years) and adolescents (10 to 19 years old) who were overweight and obese attended the consultation. All children and adolescents who consulted at the UFTM outpatient clinic were examined by the same doctor, submitted to the collection of pertinent laboratory tests, nutritional guidance provided by a nutritionist and drug treatment when necessary. During the consultation, they were invited to participate in the research project, with refusal by the same or responsible in 10 cases and exclusion from the study in 1 case due to the diagnosis of Osteogenesis Imperfecta. Thus, the final sample consisted of 161 overweight and obese children and adolescents. The eutrophic group consisted of 40 children and adolescents. A cross-sectional study was carried out.

Now the lines are written as follows:

A previous study was carried out in the city of Uberaba, Minas Gerais, Brazil from March 2012 to September 2013, where 1,125 school children and adolescents (5 to 19 years of age) were evaluated, where 916 were from public schools and 209 were from private schools; 364 participants had inadequate body weight, while 195 were overweight (17%) and 169 were obese (15%) [24].

The study participants were invited to attend the Triangulo Mineiro Federal University (UFTM) Pediatric Endocrinology Outpatient Clinic, to carry out medical consultations, laboratory tests, and receive guidance on the treatment of excess weight.

However, only 172 children (5 to 9 years) and adolescents (10 to 19 years old) attended the consultation. All 172 participants were examined by the same doctor, submitted to the collection of pertinent laboratory tests, and nutritional guidance was provided by a nutritionist along with drug treatment when necessary. During the consultation, they were invited to participate in the research project conducted from February 2013 to July 2014. Ten participants refused to participate, and we excluded one case due to the diagnosis of Osteogenesis Imperfecta. Thus, the final sample consisted of 161 overweight and obese children and adolescents. The eutrophic group consisted of 40 children and adolescents. A cross-sectional study was carried out.

2. From Table 1, under the results section you have indicated 2 groups of interest contrary to the concept of 3 groups. Please indicate the characteristics of the eutrophic group as well:

Response: Table 1, results section, was revised and we added the group of eutrophic patients as requested.

3. Include the subheadings where necessary, it is not easy to tell which paragraph has clinical implications, conclusion/summary including the limitations of the study which could be expanded:

Response: We reviewed the grammar and translation of the study to better understanding of all sections of the article. We saw no need to include subtitles.

4. Results have not been clearly communicated, the discussion has the comparison factor to other relevant literature which the majority has been cited but the logic flow is a bit missing:
Response: The discussion flow has been revised and we have added pertinent relevant literature.

Minor comments:

1. Lines 184-188, “The project was approved by the Committee of Ethics in Research with Human Beings of UFTM (protocol no. 2479). Children, adolescents and parents and/or parents were clarified about the research project and those who agreed to participate of the study project signed the term of free and informed consent and the term of assent.” Move text to somewhere under statements of ethics and revise the sentence.
   Response: The text was moved to a place about ethics statement. However, one of the guidelines received in this email was that the ethics statement should only appear in the materials and methods section. Therefore, this guidance was a little contradictory because, in the guidance sent, it asks for this phrase to appear only in the methods section and in the reviewer's comments it asks for it to appear in an appropriate place.

2. Spelling error I guess of “Eutrofic” in Figure 1. Please clarify.
 Response: Spelling error of the word eutrophic was corrected.

3. Overall the grammar in the article is not so clear to easily understand, if authors can logically simplify the text and also there are some short paragraphs which can be merged into sizeable pieces, that would be great and could improve the clarity of the article.
Response: Spelling and translation into English have been revised

4. Cite each statement that has been made in the article, as this covers authors on plagiarism. E.g. lines 67-71, 83-85. Please countercheck for others.

Response: A review of the article's references was carried out, the lines oriented for review are based on references 1; 2 e 12 that were added.
